# Does Embracing New Approaches in Homemade Fruit Spirit Production Lessen Consumer Health Risks?

**DOI:** 10.3390/toxics13060444

**Published:** 2025-05-28

**Authors:** Katarina Bijelić, Ljilja Torović, Boris Milijašević, Nebojša Kladar, Nebojša Stilinović, Branislava Srđenović Čonić

**Affiliations:** 1 Department of Pharmacy, Faculty of Medicine, University of Novi Sad, Hajduk Veljkova 3, 21000 Novi Sad, Serbia; katarina.bijelic@mf.uns.ac.rs (K.B.); nebojsa.kladar@mf.uns.ac.rs (N.K.); branislava.srdjenovic-conic@mf.uns.ac.rs (B.S.Č.); 2 Center for Medical and Pharmaceutical Investigations and Quality Control, Faculty of Medicine, University of Novi Sad, Hajduk Veljkova 3, 21000 Novi Sad, Serbia; 3 Department of Pharmacology, Toxicology and Clinical Pharmacology, Faculty of Medicine, University of Novi Sad, Hajduk Veljkova 3, 21000 Novi Sad, Serbia; boris.milijasevic@mf.uns.ac.rs (B.M.); nebojsa.stilinovic@mf.uns.ac.rs (N.S.)

**Keywords:** alcoholic beverages, distillation processes, food safety, public health, rakija

## Abstract

Fruit spirits have roots in traditions across Eastern and Central Europe. Homemade/unrecorded spirits are typically produced under inconsistent conditions, leading to inconsistent product quality. From the safety aspect, great concentration variability of exclusively harmful substances (methanol and acetaldehyde) and compounds with either favorable or hazardous properties (ethyl acetate and higher alcohols), depending on their amount, must be considered. An option to ensure their safety could be a dephlegmator, a central component in column distillation systems. To determine whether such an approach in small-scale spirit production lessens the health risks from harmful volatile compounds, 35 fruit spirits were investigated using HSS-GC-FID. Dephlegmator usage was associated with lower median methanol concentrations (1878 vs. 3723 mg/L p.a.) and a narrower concentration span. The remaining analytes showed no significant reduction in median level; however, the ranges have narrowed. A risk assessment (margin of exposure approach) revealed that dephlegmator usage increased the proportion of methanol-safe samples. The risk of acetaldehyde was equivalent or somewhat greater in the dephlegmator spirit group, suggesting challenges for optimizing the head cut during distillation. Ethyl acetate and higher alcohols did not pose a risk regardless of dephlegmator use. These findings support dephlegmator usage as a useful but insufficient intervention in home/small-scale spirit production to obtain safer products of consistent quality.

## 1. Introduction

Alcohol consumption has been a part of culture for centuries, especially in Eastern and Central European countries, occupying a central place at social gatherings, celebrations, and everyday life, making it a tradition rather than an ordinary custom. Contrary to the widespread belief that a shot of rakija benefits health, this tradition raises significant public health concerns, as evidenced by the fact that alcohol consumption has become a socially acceptable behavior. Yet, alcohol consumption is one of the leading causes of disease burden, responsible for liver cirrhosis, alcohol dependence, and other chronic diseases, particularly in Eastern and Central European countries [1,2,3]. The average per capita alcohol consumption in the WHO European Region is almost double in comparison to the global average, whereby in Central Europe, 5.79% of deaths in 2019 were attributed to alcohol use [4].

Of all the alcoholic beverages, the greatest attention is attracted by fruit spirits—“rakija” (Serbia), “palinka” (Hungary), “raki” (Albania), Țuică/Pălincă (Romania), and Slivovica/Pálenkamost (Slovakia), which are often made from plums, grapes, pears, or quinces [5]. In Serbia, plum “rakija” (slivovitz) has been listed on the UNESCO Representative List of the Intangible Cultural Heritage of Humanity in 2022, which further confirms the importance of “rakija” for the Serbian population, as it has been home-made for generations [6]. Since the production of spirits, unlike sales, by unregistered entities is not banned by law, this trend has been maintained [7]. These beverages are highly valued and play an important role in rural tourism. However, their safety is a potential issue, since the quality control of these home-made products is, at least, if present, questionable. As fruit spirit production is a matter of national pride and tradition, a balance between tradition and product safety assurance should be established [2,8,9]. People who choose to drink fruit spirits or any other alcoholic beverage consciously accept the health risks derived from ethanol consumption, but ethanol is not the only health-concerning constituent. Consumers are mostly unaware of methanol, acetaldehyde, or higher alcohols (often more abundant in unregistered than registered fruit spirits) and their associated health risks [10,11].

The quality and composition of fruit spirits are mainly influenced by the quality of the starting raw material and the length of fermentation and maturation. However, some authors point out that the type of distillation also plays an important role in spirit production, emphasizing that an adequate distillation process yields a product with desirable sensory characteristics and a lower content of harmful components [8,12,13]. One of two known types of distillation is commonly used for spirit production: alembic pot distillation or column distillation. Although both are based on the same principle, there is a notable difference in product quality.

The copper alembic pot is the most often used in home conditions and small-scale distilleries. However, since this type of distillation does not produce a fruit spirit with a desirably high ethanol content, it is necessary to conduct a double distillation, which initially requires more resources [12]. Additionally, an investigation of the profile of elements in recorded and unrecorded fruit spirits demonstrated that Cu was the most abundant element, being even fivefold higher in unrecorded spirits than in recorded ones [14]. Copper ions promote the formation of health-concerning ethyl carbamate in the distillate; a moderate or strong positive relationship between Cu content and specific fruit types was also demonstrated [10,15].

Distillation using columns with dephlegmators is somewhat more popular in industrial processes. However, nowadays, its usage in home distilleries is becoming more common, indicating the acceptance of newer approaches to obtaining better-quality products, although the health-relevant effects of small-scale rakija production employing the dephlegmator have not yet been sufficiently studied [10,12,15]. The dephlegmator is one of the most important parts of the equipment utilized for column distillation, particularly within more sophisticated installations. The advantage is a product with a high ethanol content after the first distillation. The dephlegmator plays a pivotal role in purifying the alcohol vapor fractionation by forcing some to condense, leaving only the most preferred fractions to pass the ultimate distillate. Steam reflux regulation enables the dephlegmator to influence the product’s purity and quality with minimal traces of unwanted ingredients [12,16].

Previous risk assessments from our investigation group established that unrecorded fruit spirits (produced in private homes or small-scale distilleries) from Serbia pose a higher health risk than recorded ones regarding volatile harmful compounds (methanol and acetaldehyde) [11], toxic elements (Cu and As) [14], and the process contaminant ethyl carbamate [10]. Fruit type was also established as a key factor for hazards other than ethanol [3]. These findings prompted us to look into possible interventions in spirit production directed towards lowering health-concerning compound concentrations.

Therefore, this pilot study aims to determine whether the column distillation approach, as applied in small-scale (unrecorded) spirit production, lessens the health risks posed by harmful volatile components. The chosen approach was to compare the safety profiles of unrecorded fruit spirits from Serbia produced with or without dephlegmator usage, encompassing the following steps: (1) the determination of methanol, acetaldehyde, ethyl acetate, and higher alcohol content; (2) an evaluation of compliance with legal requirements, as well as limits proposed by the AMPHORA; and (3) consumer health risk assessments.

## 2. Materials and Methods

### 2.1. Sample Information

For this research, 35 samples of unregistered (unrecorded and non-commercial) brandies (fruit spirits), namely “rakija” made from different fruits, were collected: apple (8), quince (8), grape (9), pear (3), plum (3), apricot (2), and 1 sample each of strawberry and fig. The samples were grouped based on dephlegmator usage in distillation, according to the information obtained from producers: 16 samples distilled with and 19 without it. All samples were collected in Serbia during 2024 and stored until analysis in well-sealed glass containers protected from light.

### 2.2. Sample Preparation and Analysis

Sample preparation and HSS-GC-FID (7890B GC System, Agilent Technologies, Santa Clara, CA, USA) analysis for methanol, acetaldehyde, ethyl acetate, and higher alcohol quantitative determinations were conducted according to the method previously validated and published by our investigation group [11]. An aliquot of 1 mL of calibration standard mix (acetaldehyde, ethyl acetate, methanol, isopropanol, *n*-propanol, isobutanol, *n*-butanol, and isoamyl alcohol) (purity for UV, IR, HPLC, and ACS; AppliChem Panreac ITW Companies, Darmstadt, Germany) serially diluted with deionized water (ASTM II), (Merck Millipore: Elix Essential.10 ware system, Burlington, MA, USA) or a rakija sample, 25 μL of an internal standard solution (methyl isobutyl ketone, 12.5 mg/mL), and 0.5 g of NaCl were measured in a headspace vial and analyzed using HSS/GC-FID under operating conditions, as presented in Supplementary Materials Table S1. The analytical sequence included standards for calibration verification, blanks, and a number of rakija sample duplicates to confirm method precision, as established through the method validation assay; validated method performance characteristics are presented in Table S2.

The ethanol content was determined using an alcoholometer with a thermometer by the spirit producers themselves.

### 2.3. Compliance Evaluation

The content of acetaldehyde, ethyl acetate, methanol, and higher alcohols in the analyzed rakija samples was compared with the limits defined by Regulation 2019/787 [17], which is also transposed in the regulation of the Republic of Serbia (maximum methanol content shall be 1000, 1200, or 1350 g/hL of 100% vol. alcohol (p.a.), depending on the fruit type) and the limits proposed by the International Alliance for Responsible Alcohol Consumption (AMPHORA) (acetaldehyde, methanol, and the sum of higher alcohols of 50 g/hL, 1000 g/hL, and 1000 g/hL per 100% vol. alcohol, respectively) [18,19].

### 2.4. Risk Assessment

To determine the potential risks associated with hazardous volatile compounds present in fruit brandies, the margin of exposure (MOE) approach, which was previously described in a paper published by Srđenović et al. [11], was applied. For MOE evaluations, the criteria established by the European Food Safety Authority were adopted [11,18,20]. Two scenarios of gender-specific daily alcohol consumption were employed, namely (1) average consumption (per capita consumption averaged across the entire population aged 15+) and (2) regular drinkers only (total population aged 15+ minus abstainers), according to WHO data for Serbia [21].

### 2.5. Data Processing

All obtained data were processed using Microsoft Excel (v2021) and Statistica 13.5.0.17 software (TIBCO Software Inc., Tulsa, OK, USA). Descriptive statistics (mean, median, interquartile range, and selected percentiles) were performed for the parameters of interest. The Mann–Whitney U test was used to determine the significance of differences between the concentrations of a volatile component in spirits produced using a dephlegmator or not, as well as principal component analysis (PCA).

## 3. Results and Discussion

### 3.1. Volatile Content and Compliance Assessment

**Figure 1:**
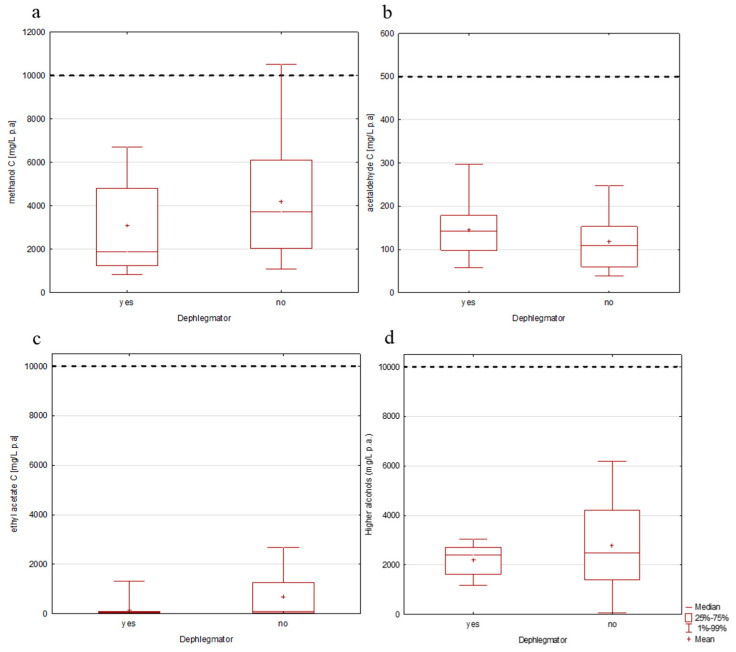
Concentrations of analytes in fruit spirits depending on whether a dephlegmator was used or not: (**a**) methanol; (**b**) acetaldehyde; (**c**) ethyl acetate; (**d**) sum of higher alcohols. ------- AMPHORA limits.

The dephlegmator-distilled spirits had a median methanol content of 1878 mg/L p.a. versus 3722 mg/L p.a. in the spirits distilled without it (Figure 1a).

Although the Mann–Whitney test did not reveal a statistically significant difference between the two groups (U = 112, *p* = 0.19), the methanol content in the non-dephlegmator group was almost double. Notably, one sample exceeded the legal limit of 10,000 mg/L p.a. for methanol, reaching 10,506 mg/L p.a., which triggers a safety alarm. This was the plum spirit sample, which corroborates previous findings that stone fruit spirits (plum and apricot) have the highest level of methanol, followed by pome fruits (apple, pear, and quince) (due to the high pectin content in both types of fruits [22]), and far behind grape rakija; no significant difference was observed for ethyl acetate and higher alcohols [3]. Therefore, it is noteworthy that the number of rakija samples distilled with and without a dephlegmator was balanced, considering fruit classes.

Furthermore, this is in line with the observations from Spaho [12], who reported that column stills with reflux can lower methanol levels by 10% in plum rakija compared to traditional pot stills, primarily due to more effective separation of volatile compounds through controlled reflux and tray design. Similarly, García-Llobodanin et al., 2011 [15], reduced methanol concentrations by roughly the same value in column-distilled pear spirits using optimized distillation parameters, particularly the reflux rate and head-cut point. Methanol is particularly difficult to remove due to its low boiling point and high water solubility, thus behaving differently than many other alcohol congeners during distillation. In pot stills, it tends to concentrate in the tail fraction as the ethanol content decreases; in column stills, it is more likely to emerge early in the distillate, where it can be effectively separated [12,22]. Despite these minor reductions, more extreme actions are required to ensure safety. As explained by Lučić [23], enzymatic treatment of the mash, acidification to inhibit pectin methylesterase activity, and premature heating of the fermented material can all minimize methanol formation during fermentation. The use of demethanolization columns during distillation was promising, with the potential to decrease methanol content by as much as 75% [24]. In addition, careful choice of yeast strains with lower methanol-forming abilities and rigorous maintenance of fermentation conditions also contribute to safer end products. It can be concluded that a dephlegmator reduces the methanol content, but it should be considered a part of a more comprehensive strategy. A combination of optimized fermentation techniques, targeted enzymatic and heat treatments, and better distillation technologies, such as demethanolization, is required to meet safety standards and reduce consumer exposure to methanol, particularly in unrecorded fruit spirits [22,25].

All spirit samples were within the EU regulatory limits and AMPHORA recommendations concerning acetaldehyde content (Figure 1b). Although the median acetaldehyde content was slightly lower in the group without a dephlegmator (109 mg/L p.a.) than in the group with it (141 mg/L p.a.), there was no statistical significance between the groups (U = 111, *p* = 0.18). Spaho [12] compared single- and double-stage pot still distillation of plum brandy and found that single-pot distillation yielded more than double the acetaldehyde content (448 mg/L p.a.) compared to double distillation (209 mg/L p.a.), underscoring the efficacy of a second run in removing volatile aldehydes. Meanwhile, García-Llobodanin et al., 2011 [15] demonstrated that, in pear spirit production, under optimized reflux and precise head-cut control, column distillation could reduce acetaldehyde by 40% relative to single-pot still distillation. These findings indicate that distillation configuration alone (pot still vs. column) does not deterministically set final acetaldehyde levels. Instead, the number of distillation stages, reflux control, vapor residence time, and the objectivity of fraction cuts are critical. A double-pot still run effectively halves acetaldehyde relative to a single run [12]. A well-controlled column can achieve similar or better reductions, albeit only if the reflux ratio and cut points are tightly regulated. Moreover, fermentation practices (yeast strain selection and anaerobic handling) and minimizing the delay between fermentation and distillation further suppress unwanted acetaldehyde formation. For small-scale producers, the practical takeaway is to combine at least two distillation passes in pot stills or employ a carefully managed column reflux using simple sensors (e.g., temperature or refractive index probes) to standardize head cuts and distill promptly after fermentation to ensure both traditional character and consumer safety [12,15,26].

When comparing the ethyl acetate concentrations (Figure 1c), no statistically significant difference between the two groups (U = 113, *p* = 0.2) was found. The median concentration for the dephlegmator group was 53 mg/L p.a. and slightly higher in the group produced without it (75 mg/L p.a.). All the values were below the regulatory limits, in line with the previously published research [3], where neither recorded nor unrecorded Serbian rakija exceeded the regulatory limit; that is, they both did not raise health concerns. Despite its comparatively low health hazard, ethyl acetate is still a key contributor to the fruit spirits’ sensory profile due to its fruit-like, sweet smell. Therefore, its presence, in acceptable amounts, is crucial to guarantee the desirable flavor qualities [8].

When the sum of higher alcohols (the total concentration of isoamyl alcohol, *n*-propanol, isobutanol, and *n*-butanol) was considered (Figure 1d), the difference between “rakija” produced with and without a dephlegmator was not statistically significant (U = 132, *p* = 0.52). The dephlegmator group ranged from 1182 to 3044 mg/L p.a., with a median concentration of 2394 mg/L p.a., while the non-dephlegmator group had a similar median of 2472 mg/L p.a. but a wider range, from 62 to 6182 mg/L p.a. The wide range in the non-dephlegmator group emphasizes the need for reflux control towards the stability of product composition. Although median content was not significantly different, the broader range and presence of outlier values in spirits produced without a dephlegmator suggest a lack of control in distillation, resulting in less uniform quality of products. Despite variations, none of the rakija samples, regardless of the production method, exceeded the AMPHORA guideline limit of 10,000 mg/L p.a. for the sum of higher alcohols. Nevertheless, from the toxicological aspect, it is important to highlight that the analysis focused only on isoamyl alcohol, *n*-propanol, isobutanol, and *n*-butanol; other fusel alcohols, which could contribute to the content of higher alcohols, such as 2-methyl-1-butanol (amyl alcohol), 1-hexanol, 2-phenylethanol, and others commonly found in fruit spirits, were not included in the quantification [27]. However, it is essential to note that even in permissible amounts, higher alcohols can still affect spirit sensory quality, especially when present in mutually disproportionate concentrations. Unpleasant sensory effects such as pungency and burning mouthfeel were previously shown for spirits from home distilleries with elevated levels of fusel alcohols. Thus, while the dephlegmator may not significantly reduce the absolute levels of higher alcohols, its use promotes product compositional consistency and flavor compound composition. In small-scale or traditional distillation practices, this can improve both the sensory quality and consumer safety at once [28,29].

**Figure 2:**
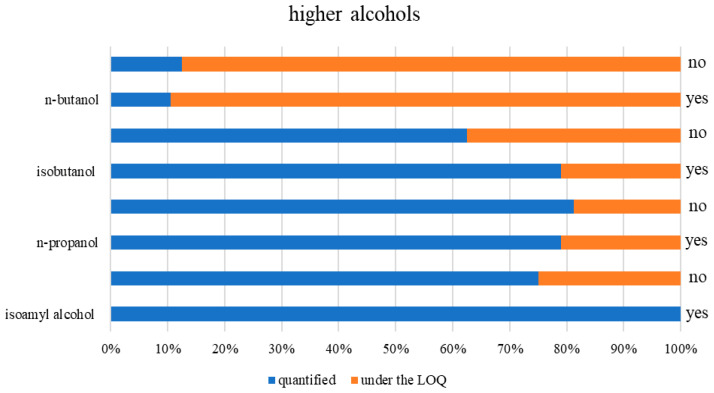
Percentage of quantified higher alcohols in fruit spirit samples (yes—dephlegmator used; no—dephlegmator not used).

Among the higher alcohols detected, isoamyl alcohol was the most abundant across all analyzed spirit samples (Figure 2), as it was quantified in 89% of the samples. Isoamyl alcohol was previously identified as the dominant fusel alcohol in fruit spirits, deriving from the amino acid leucine via the Ehrlich pathway [29]. In samples produced using a dephlegmator, isoamyl alcohol ranged from 808 to 2960 mg/L p.a., while the non-dephlegmator group showed a broader range, from 1339 to 5124 mg/L p.a., suggesting less consistent separation of volatile compounds. However, the median contents were similar at 1904 mg/L and 2082 mg/L p.a. (U = 128, *p* = 0.44). The observed difference in concentration spans aligns with prior studies showing that column stills with dephlegmators provide more controlled separation of volatiles via rectification and reflux, while pot stills (often used without dephlegmators) may retain more fusel alcohols due to less precise cut points [12].

*n*-Propanol, which was quantified in 80% of all analyzed spirits (Figure 2), was ranked second when considering the abundance of higher alcohols. In the dephlegmator group, its concentration ranged from 8 to 694 mg/L p.a. (median: 304 mg/L p.a.), while in the group without it, the range was from 24 to 1230.48 mg/L p.a. (median: 279.33 mg/L p.a.). Again, no significant difference between medians was observed (U = 131, *p* = 0.5), although the variability was much greater in the absence of a dephlegmator, a pattern also noted in previous chemometric assessments of fruit spirits [3].

Isobutanol, which was present in 71% of all samples (Figure 2), had a median of 43 mg/L p.a. (range: 27–413 mg/L p.a.) in the dephlegmator group and 33 mg/L p.a. (range: 25–427.78 mg/L p.a.) in the non-dephlegmator group (U = 126, *p* = 0.39, non-significant). On the other hand, *n*-butanol was the least frequently detected higher alcohol, appearing in only four samples: the dephlegmator group yielded values of 111 and 166 mg/L p.a.; the non-dephlegmator group had 96 and 588 mg/L p.a. Due to the limited number of samples, no statistical comparison was conducted, but the isolated high reading in the non-dephlegmator group confirms product compositional variability.

The maximum allowable concentrations of individual long-chain alcohols in alcoholic beverages are not regulated, although they may contain significant amounts, depending on the distillation method/type of beverage. Although the safety of chronic exposure is not fully established for any of them, it is known that the acute toxicity of straight-chain monohydroxy alcohols is directly proportional to the carbon chain length (Richardson’s law). Their presence is only indirectly monitored by distillers or official laboratories, in line with the provision for the sum of higher alcohols, as they are meant to maintain the sensory quality of the final product. Mukerjee and Siciliano proposed that concentrations of alcohols in commercial beverages without labeling should be limited to a maximum of 86 mM multiplied by 0.344 for each additional carbon compared to ethanol. For *n*-propanol, that would be 1774 mg/L p.a., and for *n*-butanol, that would be 754 mg/L p.a. The content of straight-chain monohydroxy alcohols in all investigated samples was below or, in sporadic cases, near the proposed limit [30].

**Figure 3:**
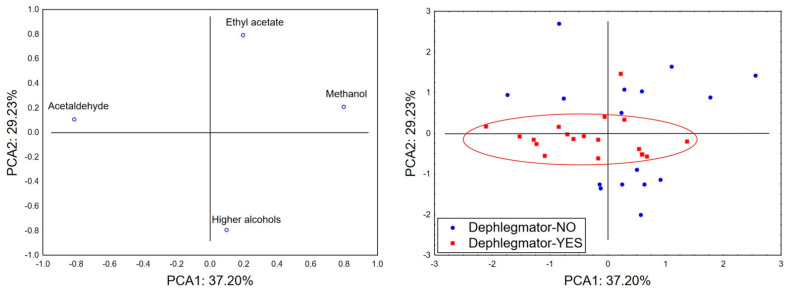
Principal component analysis for potentially harmful volatile substances in fruit spirits: principal component loadings and the position of analyzed spirits in the space defined by PCA1 and PCA2.

The application of principal component analysis (PCA) on data reporting the concentrations of methanol, acetaldehyde, ethyl acetate, and the sum of higher alcohols in the analyzed samples suggests that the first two principal components (PCA1 and PCA2) are of statistical significance for defining a simplified model of the original dataset (determined according to the criteria of the scree plot and eigenvalues (Ev; Ev > 1)—Ev (PCA1) = 1.49 and Ev (PCA2) = 1.17)). Namely, PCA1 and PCA2 were found to describe around 66% of the original dataset’s variability (Figure 3), whereas the size of the samples’ variability (in terms of PCA1) mostly correlates with the concentrations of acetaldehyde and methanol. The position of the analyzed samples in the space defined by the first two principal components indicates a trend of lower methanol concentrations in samples produced via dephlegmator application. On the other hand, no specific patterns of samples’ separative grouping could be noticed in terms of PCA2, which correlates with the levels of ethyl acetate and the sum of higher alcohols. However, a more “compact” grouping of samples (Figure 3, red circle) produced by the application of a dephlegmator in terms of PCA2 suggests the lower variability of ethyl acetate and higher alcohol concentrations in these samples.

To conclude, although a dephlegmator did not significantly reduce the absolute concentration of individual higher alcohols or ethyl acetate, it appeared to contribute to a more uniform product composition with fewer extremes. In small-scale settings, where quality consistency is a challenge, even modest improvements in fractionation control could enhance the safety and organoleptic quality of the final spirit.

### 3.2. Risk Assessment

**Figure 4:**
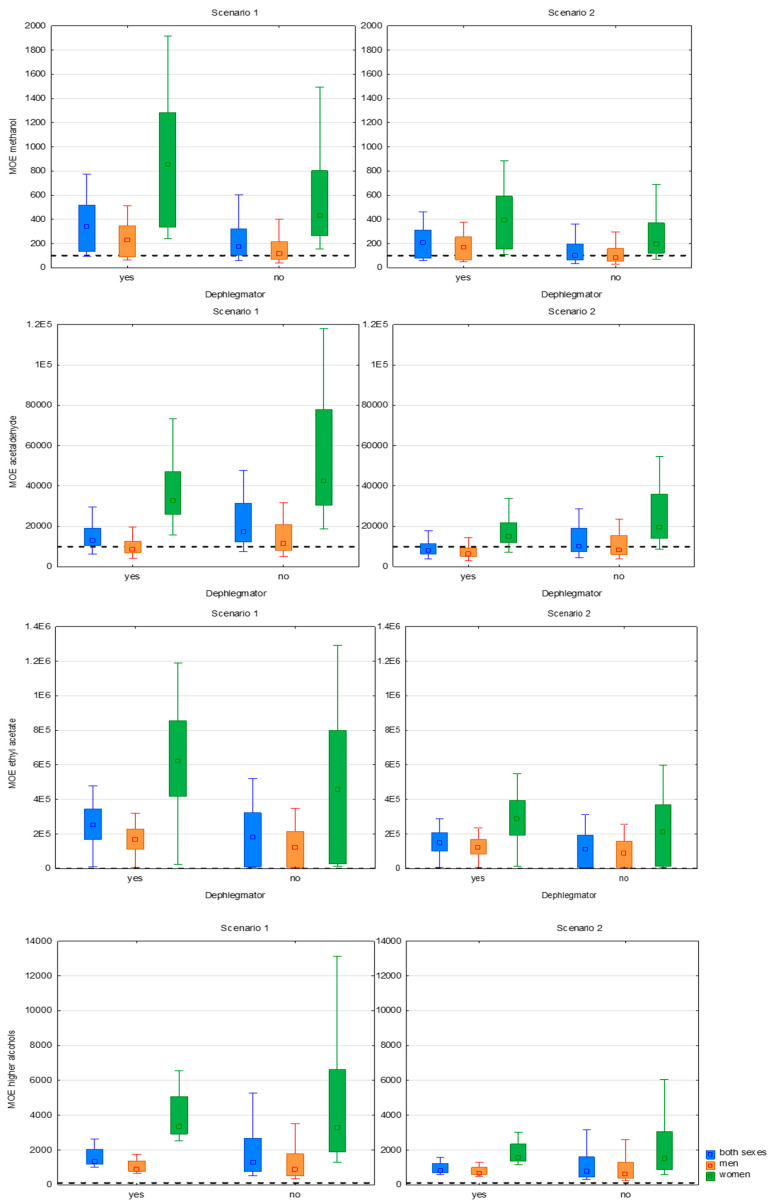
Margins of exposure for methanol, acetaldehyde, ethyl acetate, and higher alcohols depending on whether or not a dephlegmator was used; -------- safety margin (100 for methanol, 10,000 for acetaldehyde, and 100 for ethyl acetate and higher alcohols); □ mean; ▯ 25–75%; I 1–99%.

**Figure 5:**
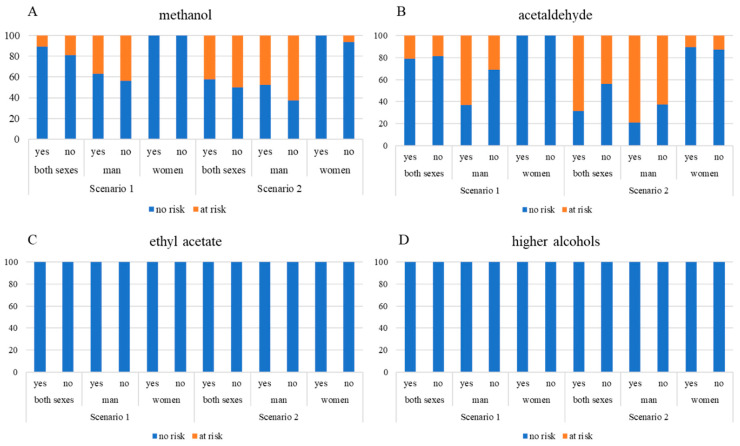
Percentage of fruit spirits posing risk/no risk; (**A**) methanol; (**B**) acetaldehyde; (**C**) ethyl acetate; (**D**) higher alcohols.

To assess the influence of the distillation technique on consumer safety, the MOE approach was applied to evaluate the methanol-related risk in unrecorded fruit spirits (Figure 4). In Scenario 1, where exposure levels are equated to general population averages, a higher share of samples distilled with a dephlegmator was in the “no risk” category than in the case of spirits distilled without it. Among male consumers, 63% of dephlegmator-distilled spirits did not pose a risk (MOE > 100), compared to 56% in the non-dephlegmator group; among females, no samples at all posed risk concerns due to lower ethanol consumption volumes (Figure 4 and Figure 5).

In Scenario 2, where exposure estimates reflect habitual or high-end consumption, the distinction between the two distillation methods was more pronounced in favor of spirits distilled with the dephlegmator, especially in the case of male consumers: 53% vs. 37% fell into the no-risk category (Figure 5); for females, the proportion was 100:93% (Figure 5). These findings lead to the conclusion that a dephlegmator may reduce the health risk associated with methanol, particularly among regular male drinkers. The lower incidence of concerning MOE values in the dephlegmator group aligns with prior chemical analyses showing lower methanol concentrations, albeit without statistical significance between the medians of the two groups.

Moreover, previous risk assessments in unrecorded spirits from Serbia by Srdjenović-Čonić, Kladar, Božin, and Torović [11] highlighted methanol as one of the main risks to consumer health, with MOE values often falling below the safety margin. The current data confirm this pattern but also show that improvements in distillation technology, such as dephlegmator incorporation, can help shift a larger share of products above the minimum acceptable MOE value, especially in populations with elevated alcohol consumption. Altogether, these results demonstrate that while methanol remains a key toxicological concern in unrecorded spirits, its impact on consumer safety can be mitigated through technical upgrades in production, particularly those enhancing reflux control and volatile separation [11,12,15]

When considering the influence of a dephlegmator on the health risk associated with acetaldehyde, the results of the MOE-based assessment revealed that this distillation feature did not offer a clear protective effect. In Scenario 1 (Figure 4 and Figure 5), more than two-thirds (68%) of the spirits distilled without a dephlegmator fell into the “no risk” group for male consumers, in contrast to one-third (36%) of those from the dephlegmator group; there were no risky samples in the case of females. The difference remained at approximately the same level under Scenario 2, 37% vs. 21% for males; for females, there was no practical difference (almost 90% of the samples from each group). This finding indicates that dephlegmator usage could not remove the acetaldehyde effectively and might even hinder its removal in some cases. The explanation can be found in the physicochemical properties of acetaldehyde, since it has a low boiling point and consequently concentrates in the head fraction. If head–heart separation is not optimally controlled, acetaldehyde can pass into the heart cut even when modern equipment is employed. Some studies have shown that column distillation without well-defined fractionation settings results in higher levels of acetaldehyde, while more traditional pot stills based on slower, sensory-guided cuts can allow for greater separation. Thus, even equipment with greater technical potential may not guarantee a safer product without adequate operational expertise [11,12,15,31].

The risk assessment for ethyl acetate and higher alcohols indicates that neither analyte posed a health risk to consumers, regardless of the distillation method or the consumption scenario. Across all categories, both sexes, men and women, in both scenarios (1—average consumption, 2—regular drinkers), 100% of spirits were placed in the “no risk” category (Figure 4 and Figure 5). This finding is consistent with previous studies on fruit spirits, which have shown that although higher alcohols and ethyl acetate contribute significantly to the aroma and flavor profile of the beverage, they are generally not considered critical toxicological hazards at levels typically found in traditional distillates [3,11]. Regardless of the rigidness of production condition control, these compounds are typically in the safe zone, especially when compared to substances like methanol or acetaldehyde, which are commonly identified as major hazards. However, their role in the sensory perception and acceptability of the final product remains important and warrants attention in quality evaluations [29].

Finally, some study limitations need to be noted. The findings rely on a rather small collection of rakija samples, lacking information about yeast strains and the length of fermentation or the distillation temperature applied in the production process, which could also affect the volatile profile of the spirits. However, since the study has a comparative approach, it is a reasonable assumption that the balancing of the samples in terms of their botanical origin and producers versus two technologically distinctive production processes compensates the effect of the mentioned factors to an extent, ensuring that observed variability is under the dominant influence of dephlegmator employment in the distillation process.

This pilot research, together with several previous ones, indicates the importance of a deeper understanding of the distillation process and thus warrants further research in this area.

## 4. Conclusions

This pilot study explored the impact of a dephlegmator on volatile compound concentrations in unregistered rakija and the associated risk arising from its consumption. Although the dephlegmator did not reduce the absolute concentrations of acetaldehyde, ethyl acetate, or the higher alcohols to any significant extent, it did have a clear tendency to lower methanol concentrations and increase the consistency of the final product’s chemical profile. Spirits produced with a dephlegmator showed narrower concentration ranges of the compounds of interest and fewer outlier values, particularly in the case of methanol and higher alcohols, reflecting improved fractionation control.

Methanol remains the most toxicologically relevant compound in unrecorded spirits, as well as in regular drinkers. A dephlegmator greatly increased the percentage of samples within the “no risk” group, especially for regular male consumers. The exception was acetaldehyde; risks were more marked in the dephlegmator group, presumably resulting from initial vapor passage and improper head-cut separation. Higher alcohols and ethyl acetate were not a notable health concern.

The study findings support a dephlegmator as a useful option in home-made or small-scale spirit production to improve product safety and chemical consistency. Nevertheless, technology alone is insufficient. Thus, proper training in distillation management, fermentation condition optimization, and the use of complementary techniques, such as enzymatic treatment and demethanolization, is necessary to ensure consistent product quality and minimize health risks associated with fruit spirit consumption.

## Data Availability

The original contributions presented in this study are included in the article/Supplementary Materials. Further inquiries can be directed to the corresponding author.

## Supplementary Materials

The following supporting information can be downloaded at https://www.mdpi.com/article/10.3390/toxics13060444/s1, Table S1. HSS/GC-FID instrument operating conditions; Table S2. HSS/GC-FID method performance characteristics and the dataset.
