# Supplementary material for: Does Embracing New Approaches in Homemade Fruit Spirit Production Lessen Consumer Health Risks?
Source: Toxics. 2025 May 28;13(6):444. doi: 10.3390/toxics13060444 (PMC12197528; doi:10.3390/toxics13060444)
Supplement: Supplementary file 1 [file toxics-13-00444-s001.zip › toxics-3633663-supplementary.pdf]

Supplementary Material

Does Embracing New Approaches in Homemade Fruit Spirit Production Lessen Consumer Health Risks?

Katarina Bijelić [1,2], Ljilja Torović [1,2,*], Boris Milijašević [3], Nebojša Kladar [1,2], Nebojša Stilinović [3] and Branislava Srđenović Čonić [1,2]

[1] Department of Pharmacy, Faculty of Medicine, University of Novi Sad, Hajduk Veljkova 3, 21000 Novi Sad, Serbia; katarina.bijelic@mf.uns.ac.rs (K.B.); nebojsa.kladar@mf.uns.ac.rs (N.K.); branislava.srdjenovic-conic@mf.uns.ac.rs (B.S.Č.)
[2] Center for Medical and Pharmaceutical Investigations and Quality Control, Faculty of Medicine, University of Novi Sad, Hajduk Veljkova 3, 21000 Novi Sad, Serbia
[3] Department of Pharmacology, Toxicology and Clinical Pharmacology, Faculty of Medicine, University of Novi Sad, Hajduk Veljkova 3, 21000 Novi Sad, Serbia; boris.milijasevic@mf.uns.ac.rs (B.M.); nebojsa.stilinovic@mf.uns.ac.rs (N.S.)
* Correspondence: ljilja.torovic@mf.uns.ac.rs

		**Page**
**Table S1**	HSS/GC-FID instrument operating conditions	2
**Table S2**	HSS/GC-FID method performance characteristics	2
	Dataset	3

Table S1. HSS/GC-FID instrument operating conditions

**HSS/GC-FID instrument:** 7890B GC System, Agilent Technologies				
Oven temperature program				
	Rate (°C/min)	*Value (°C)*	*Hold time (min)*	*Run time (min)*
(Initial)	-	30	5	5
Ramp 1	20	100	0	8.5
Ramp 2	35	200	0	11.4
**Parameter**			**Operating conditions**	
**Headspace sampler**				
Vial pressurization gas:			Helium	
Oven temperature:			90°C	
Loop temperature:			115°C	
Transfer line temperature:			120°C	
Vial equilibration:			10 min	
Injection duration:			0.10 min	
GC Cycle time:			25 min	
**GC**				
Inlet:			200°C, 75:1 split ratio in split mode	
Helium carrier gas flow rate:			2.5 mL/min, constant flow mode	
**FID**				
Heater:			280°C	
H2 Flow:			40 mL/min	
Air Flow			450 mL/min	
Makeup Flow			50 mL/min	
Run time:			11.4 min	

Table S2. HSS/GC-FID method performance characteristics

Analyte	Concentration range (mg/L p.a.)	Calibration equation	R^2	Accuracy* (recovery, %) (n=3)	Precision RSD (%)		LOQ (mg/L p.a.)
					Intra-day precision (n=3)	Inter-day precision (n=3)	
acetaldehyde	48.75-975	y = 0.0016x + 0.0348	0.9984	87.5-109.6	9.48	6.64	48.75
ethyl acetate	18-1800	y = 0.0027x + 0.0211	0.9972	85.3-105.4	6.53	5.07	18
methanol	98.75-9875	y = 0.0005x + 0.0381	0.9995	89.9-114.6	7.33	4.66	98.75
n-propanol	12.5-1250	y = 0.0010x + 0.0161	0.9993	86.6-114.3	6.68	4.98	12.5
iso-butanol	12.5-1250	y = 0.0013x + 0.0198	0.9985	90.7-110.4	5.90	5.35	12.5
n-butanol	62.5-1250	y = 0.0009x + 0.0219	0.9919	93.0-106.8	7.68	5.19	62.5
iso-amyl alcohol	37.5-3750	y = 0.0008x + 0.0281	0.9992	96.5-112.1	6.84	4.83	37.5

*Accuracy range based on low, medium and high concentration recovery checks (standard addition in 45% aq. ethanol); R^2-correlation coefficient; LOQ-limit of quantification.

Dataset

Sample	Dephlegmator	Methanol [mg/L p.a]	Acetaldehyde [mg/L p.a]	Ethyl acetate [mg/L p.a]	*n*-propanol [mg/L p.a]	*n*-butanol [mg/L p.a]	Isobutanol [mg/L p.a]	isoamyl alcohol [mg/L p.a]
1	yes	4290.49	89.10	28.15	366.54	31.25	6.25	2305.95
2	yes	1878.41	141.66	46.70	4.28	111.95	6.25	1937.23
3	yes	4625.88	145.72	56.68	8.12	31.25	93.97	1049.33
4	yes	4798.04	102.94	42.33	2.54	31.25	28.78	2793.99
5	yes	1481.93	139.32	50.54	223.51	31.25	41.64	1482.26
6	yes	4964.21	143.51	1303.17	6.25	31.25	6.25	1325.53
7	yes	837.06	133.66	71.05	329.31	31.25	6.25	1001.16
8	yes	1760.32	297.84	136.46	488.12	31.25	56.92	1782.09
9	yes	897.50	178.98	58.71	8.86	31.25	43.63	2960.52
10	yes	1503.10	231.50	76.10	423.84	31.25	34.75	1904.60
11	yes	965.94	190.05	80.25	281.63	31.25	413.08	1823.00
12	yes	6712.07	168.93	53.46	694.30	31.25	66.13	808.74
13	yes	1111.61	98.48	29.74	304.59	31.25	61.24	1209.80
14	yes	3659.21	63.43	32.76	324.74	31.25	33.02	2273.93
15	yes	5303.00	120.30	44.56	6.25	31.25	27.88	2642.37
16	yes	1469.25	207.02	96.99	422.15	31.25	61.28	1965.69
17	yes	4621.63	173.28	85.44	426.42	31.25	83.37	1989.23
18	no	10506.77	44.65	1268.05	1230.48	31.25	39.43	18.75
19	yes	6492.75	58.01	34.13	417.62	166.17	68.02	1443.88
20	no	3346.77	82.36	34.89	155.87	31.25	6.25	3210.64
21	no	2466.44	52.27	25.88	3.50	31.25	48.81	3811.44
22	no	7155.10	54.10	1394.31	716.28	31.25	173.45	1526.48
23	yes	1249.02	89.48	39.06	209.26	31.25	69.86	2394.64
24	no	6651.49	211.53	41.49	1032.47	31.25	36.77	3947.83
25	no	5913.27	115.97	48.20	363.21	31.25	6.25	3898.72
26	no	4971.66	149.12	62.76	996.20	31.25	30.21	5124.80
27	no	1255.38	103.82	40.98	6.25	588.24	6.25	3687.88

Dataset - *continue*

Sample	Dephlegmator	Methanol [mg/L p.a]	Acetaldehyde [mg/L p.a]	Ethyl acetate [mg/L p.a]	*n*-propanol [mg/L p.a]	*n*-butanol [mg/L p.a]	Isobutanol [mg/L p.a]	isoamyl alcohol [mg/L p.a]
28	no	3561.29	118.47	1602.82	246.30	31.25	146.69	2073.67
29	no	4627.42	111.22	46.59	679.20	31.25	62.20	18.75
30	no	1389.52	107.00	42.25	220.65	31.25	427.78	3441.80
31	no	1752.27	235.55	87.37	312.37	31.25	6.25	18.75
32	no	6261.38	66.45	951.82	6.25	31.25	6.25	18.75
33	no	1073.14	156.53	1268.58	143.99	31.25	48.21	2092.27
34	no	2323.79	39.39	1263.98	24.90	31.25	25.78	1382.70
35	no	3883.99	247.73	2679.48	432.47	96.74	6.25	1339.90
